# Comparative analysis of *β*-glucan, phenolic compounds, and targeted metabolomics in whole-grain highland barley varieties: Effects of cooking-induced changes

**DOI:** 10.1016/j.fochx.2025.102675

**Published:** 2025-06-18

**Authors:** Xinkun Wang, Chune Peng, Jingwen Fan, Hongyu Si, Sujun Sun, Xiaodan Dong, Hengzhen Wang, Yueming Wang, Peng Deng, Lizeng Peng

**Affiliations:** a Key Laboratory of Novel Food Resources Processing / Institute of Agro-Food Sciences and Technology, Shandong Academy of Agricultural Sciences, Jinan 250100, China; b Energy Research Institute, Qilu University of Technology (Shandong Academy of Sciences), Jinan 250014, China

**Keywords:** Anthocyanins, Β-Glucan, Antioxidants, Metabolomics, Cooking methods, Barley varieties

## Abstract

This study examines the phytochemical profiles and antioxidant activities of highland barley varieties with white, blue, and black seed coat colors, focusing on the effects of cooking methods on black barley. Among the varieties, black barley, particularly Xiongzhang type, showed high concentrations of anthocyanins, proanthocyanidins, flavonoids, and phenolics, all of which are associated with enhanced antioxidant activities. Metabolomic profiling revealed significant biochemical diversity across the barley samples closely linked to seed coat color. Cooking methods substantially influenced *β*-glucan levels and metabolomic profiles. Specifically, frying and baking increased *β*-glucan content while inducing specific metabolic changes. The findings highlight the nutritional advantages of black barley and the role of cooking techniques in preserving its bioactive compounds. This study provides valuable insights for breeding initiatives and dietary recommendations to enhance the nutritional quality and health benefits of barley.

## Highlights

• Black barley exhibited enhanced antioxidant activity.
• Metabolomic diversity was strongly linked to seed coat color.
• Cooking methods significantly influenced β-glucan content and metabolome.

## 1 Introduction

Highland barley (*Hordeum vulgare* L. var. nudum), also known as hulless or naked barley, is a distinctive crop traditionally cultivated in the high-altitude regions of Tibet, Qinghai, and other parts of the Himalayas. Resilient to harsh environmental conditions, it is a vital part of the diet and culture in these areas due to its nutritional benefits (Obadi, Qi, & Xu, 2021). With growing interest in the health-promoting properties of highland barley, understanding its phytochemical composition and the effects of processing methods on these components are essential. Such insights could support the expanded use of highland barley in functional foods and nutraceuticals.

Highland barley is renowned for its high dietary fiber content, particularly *β*-glucan, a soluble fiber associated with numerous health benefits. *β*-Glucan helps reduce blood cholesterol, improves glycemic control, and supports immune function (Zeng et al., 2020). Studies show that *β*-glucan from highland barley can significantly lower low-density lipoprotein cholesterol and total cholesterol levels without affecting high-density lipoprotein cholesterol (Obadi, Sun, & Xu, 2021). Additionally, *β*-glucan intake has been linked to increased insulin sensitivity and a reduced risk of type 2 diabetes (Tosh, 2013). Given these properties, highland barley serves as a valuable dietary component for supporting cardiovascular health and managing metabolic disorders.

In addition to being rich in *β*-glucan, highland barley contains a wide range of phenolic compounds that are potent antioxidants (Goudar, Sharma, Janghu, & Longvah, 2020). These phenolic compounds include phenolic acids, flavonoids, and anthocyanins, and substantially enhance the antioxidant capacity of highland barley and offer protective effects against oxidative stress-related diseases, such as cardiovascular disease, cancer, and neurodegenerative disorders (Scalbert, Manach, Morand, Rémésy, & Jiménez, 2005). By neutralizing free radicals, these compounds reduce oxidative stress and inflammation, which are key factors in the development of many chronic diseases. Responsible for the blue and black pigmentation of highland barley grains, anthocyanins are particularly noted for their anti-inflammatory and anticarcinogenic properties (Tsuda, 2012). These pigments not only enhance the visual appeal of the grains but also contribute to their health benefits, making highland barley an appealing ingredient for health-oriented food products.

The seed coat color of barley grains is closely correlated with their phytochemical composition and antioxidant activity (Dang, Zhang, Zhang, Yang, & Xu, 2022). Compared to white varieties, black and blue barley varieties are richer in anthocyanins and typically exhibit higher antioxidant activity (Kofuji et al., 2012; Xia et al., 2024). These variations in seed coat color result from genetic differences that influence the types and concentrations of phytochemicals within the grains. The genetic and biochemical diversity associated with seed coat color highlights the potential of these varieties for developing functional foods with enhanced health benefits (H. Zhao et al., 2022). For instance, with its relatively high levels of anthocyanins and other phenolic compounds, black highland barley could be beneficial in formulations aimed at boosting antioxidant intake. Understanding the relationship between seed coat color and phytochemical content can guide the selection of barley varieties for specific health applications and nutritional interventions.

Cooking methods significantly influence the nutritional and phytochemical profiles of highland barley (Wang et al., 2023). Thermal processing can modify the solubility and bioavailability of dietary fibers and phytochemicals, thereby influencing their health benefits(C. Zhao et al., 2019). In particular, steaming and baking can enhance the extraction and solubilization of *β*-glucan, increasing its availability and potential health benefits. In contrast, frying and germination may degrade *β*-glucan and other sensitive compounds, reducing their effectiveness (Y.-P. Bai, Zhou, Zhu, & Li, 2021). Depending on the cooking method, these alterations can either enhance or reduce the health-promoting properties of highland barley. Understanding these effects is essential for optimizing cooking methods to preserve or boost the nutritional quality of highland barley.

The aim of this study was to 1) compare the phytochemical profiles and antioxidant activities of highland barley varieties with different seed coat colors, 2) investigate the impact of various cooking methods on *β*-glucan content and metabolomic profiles in black highland barley and 3) identify the distinct metabolic pathways affected by seed coat color and cooking methods via Kyoto Encyclopedia of Genes and Genomes (KEGG) pathway enrichment analysis. By achieving these objectives, this study aims to provide valuable insights into the nutritional and functional properties of highland barley. This knowledge may support the development of functional foods and dietary guidelines that maximize the health benefits of highland barley.

## 2 Material and methods

### 2.1 Plant samples

A total of nine barley samples were used in this study, all of which were provided by the Biotechnology Research Institute of the Shigatse City Science and Technology Bureau in Tibet. These samples included seven local varieties and two cultivated varieties, categorized by color as follows: white (Xila22, Zangqing2000, and Gamuguori), blue (Youxi, Quduigamu, and Garu), and black (Sagui, Qujia, and Xiongzhang). The highland barley grains were cleaned and dried in an oven at 50 °C for 12 h, grounded to pass through a 60-mesh screen, and stored at −20 °C for further use.

### 2.2 Cooking

The barley grains were subjected to various traditional cooking methods, including frying, steaming, baking, and germination, with untreated grains serving as a control.

Frying: The barley grains were fried using the traditional Tibetan tsampa preparation method. The grains were first selected, cleaned, and dried. In this process, a specific amount of barley was fried in a pan containing fine sand. Once the sand temperature reached 100 °C, the barley grains popped at a rate of over 90 %. The barley and sand were then separated using a sieve.

Steaming: The barley grains were steamed according to a traditional method for preparing steamed buns. After selection, cleaning, and drying, the grains were ground and sifted through an 80-mesh sieve. A flour-to-water ratio of 5:4 (g:g) was then shaped and steamed for 30 min until fully cooked.

Baking: The barley grains were baked following a traditional method for making cookies. After selection, cleaning, and drying, the grains were ground and passed through an 80-mesh sieve. Water was added to form a dough, which was molded and baked in an oven at 180 °C with top and bottom heat for 15 min until fully cooked.

Germination: The selected and cleaned barley grains were placed on specialized germination trays and incubated in a growth chamber at 25 °C for 4 days. After this germination period, the grains were harvested, dried, ground, and passed through a 60-mesh sieve. The samples were then stored at −20 °C for further analysis.

### 2.3 Β-Glucan content

Beta-glucans were measured in highland barley and various food products using an enzymic method known as AOAC Method 995.16 (McCleary & Mugford, 1997), “B-D-Glucan in Barley and Oats, Streamlined. Enzymatic Method”.

### 2.4 Anthocyanin content

The anthocyanin content was determined following the method described in a previous study (Zhu et al., 2015) with minor modifications. One gram of barley flour was mixed with 20 mL of ethanol solution (pH 3.0, 60 % *w*/w), stirred thoroughly, and extracted by shaking at 120 rpm at 40 °C for 1 h. The mixture was then centrifuged at 14816*g* for 15 min. Two aliquots of the supernatant (1 mL each) were adjusted to a final volume of 5 mL using potassium chloride buffer (pH 1.0) and sodium acetate buffer (pH 4.5). After allowing the solution to stand for 1 h, the absorbance was measured at 515 nm and 700 nm. The anthocyanin content was calculated using the formula: Anthocyanin content = (A1.0 - A4.5) × MW × 1000/(ε × C), where A1.0 and A4.5 are the absorbance values at 515 nm and 700 nm for the samples in pH 1.0 and pH 4.5 buffers, respectively. MW is the molecular weight of Cyanidin 3-O-glucoside (C3G) (449.2), ε is the extinction coefficient of C3G (26,900), and C is the buffer concentration.

### 2.5 Proanthocyanidin content

The proanthocyanidin content was measured according to the method described in a previous study (Hümmer & Schreier, 2008). One gram of barley flour was mixed with 25 mL of pure methanol and shaken in the dark at 120 rpm for 1 h. The mixture was centrifuged at 14816*g* for 15 min, and the supernatant was collected. For analysis, 1 mL of the sample solution was mixed with 3 mL of 4 % vanillin-methanol solution, followed by addition of 1.5 mL of concentrated hydrochloric acid. The solution was mixed thoroughly and left to stand at 22 °C for 10 min. A calibration curve was constructed using catechin standard solutions at gradient concentrations. Absorbance (A) was measured at 500 nm against a reagent blank. Linear regression yielded the equation: A = 5.3012C + 0.0023 (R^2^ = 0.999), where C is the catechin concentration (mg/mL). Proanthocyanidin content in barley samples was quantified against this curve and expressed as mg per 100 g DW.

### 2.6 Total flavonoid content

The total flavonoid content was determined following the method described in a previous study (He, Liu, & Liu, 2008) with minor modifications. Five grams of barley flour were combined with 100 mL of 50 % ethanol solution and extracted by shaking at 120 rpm at 40 °C for 2 h. The mixture was centrifuged at 14816*g* for 15 min, and the supernatant was collected and concentrated to a volume of 10 mL under reduced pressure. To proceed with the analysis, 1 mL of the concentrated flavonoid solution was mixed with 1 mL of a 5 % sodium nitrite solution, shaken, and allowed to stand for 5 min. Next, 2 mL of 10 % aluminum nitrate solution was added, and the mixture was shaken and left to stand for 1 h. Finally, 2 mL of 1 mol/L sodium hydroxide solution was added, and the volume was adjusted to 10 mL using 30 % ethanol. Rutin was used as a standard to create the calibration curve: A = 21.332C + 0.0043(R^2^ = 0.999)The total flavonoid content was calculated using the formula: ω = (m_1_ × V)/(M × V_1_) × 100. Where m_1_ represents the total flavonoid mass of the sample solution, calculated from the standard curve (in mg); V is the extraction volume of the sample (in mL); V1 is the volume of the sample used for measurement (in mL); and M is the mass of the sample (in g).

### 2.7 Total phenolic content

Two grams of barley flour were combined with 20 mL of precooled 80 % acetone solution, mixed thoroughly, and extracted by shaking at 120 rpm under nitrogen for 1 h. The mixture was then centrifuged at 14816*g* for 15 min, and the supernatant was collected. This residue was subjected to two additional extractions, after which all supernatants were combined and concentrated under reduced pressure at 45 °C using a rotary evaporator until dry. To obtain the bound phenolic extract, the remaining barley flour residue was digested with 10 mL of 2 mol/L sodium hydroxide solution for 1 h, neutralized with 2 mol/L hydrochloric acid, and defatted three times with n-hexane. The solution was extracted with 20 mL of ethyl acetate under nitrogen at 120 rpm for 1 h and centrifuged at 14816*g* for 15 min with the supernatant subsequently collected. This residue was subjected to five additional extractions, and the combined supernatants were concentrated under reduced pressure at 45 °C until dry. The phenolic content in both the free and bound phenol extracts was determined using the Folin-Ciocalteu method. Specifically, 100 μL of the sample extract was combined with 400 μL of distilled water and 100 μL of Folin-Ciocalteu reagent, shaken and left to react for 6 min. Then, 1 mL of 7 % sodium carbonate solution and 1 mL of ultrapure water were added. The mixture was shaken and allowed to stand in the dark for 90 min before measuring the absorbance at 760 nm. The phenolic content was calculated using a standard curve established with gallic acid (A = 0.0046C + 0.0054，R^2^ = 0.999), with the total phenolic content determined as the sum of the free and bound phenolic contents. The total phenolic content was calculated using the formula:W = c × V × N/M*.* Where W is the content of total polyphenols in barley samples, mg/kg; M is the mass of barley sample (g); c is the mass concentration of barley sample (μg/mL); V is the volume of the extracted liquid (mL); N is the dilution factor.

### 2.8 Oxygen radical absorption capacity (ORAC) assay

The ORAC was measured according to the method described in a previous study (Wolfe & Liu, 2008) with some modifications. One gram of barley flour was combined with 25 mL of precooled ethyl acetate and extracted by shaking at room temperature in the dark for 1 h. The mixture was filtered through Whatman filter paper, and the filtrate was collected. This residue was subjected to two additional extractions, and the filtrates were combined and evaporated to dryness under reduced pressure at 35 °C. The fraction was redissolved in 80 % ethanol to obtain the crude extract. The crude extract and Trolox standard were diluted with 75 mmol/L phosphate buffer (pH 7.4) to the appropriate concentrations. To each well of a 96-well fluorescence plate, 20 μL of the sample or Trolox standard solution was added, followed by 20 μL of 75 mmol/L phosphate buffer (pH 7.4) and 20 μL of 70 nmol/L fluorescein sodium solution. The plate was incubated at 37 °C for 20 min. The reaction was initiated by adding 140 μL of 12 mmol/L 2,2′-azobis(2-methyl-propanimidamide) dihydrochloride solution. Fluorescence intensity was measured continuously at an excitation wavelength of 485 ± 20 nm and an emission wavelength of 530 ± 20 nm every 2 min until the fluorescence decayed to the baseline (D. Huang, Ou, Hampsch-Woodill, Flanagan, & Prior, 2002). The ORAC value of the sample was calculated using a standard curve (A = 0.9809C + 1.0345, R^2^ = 0.9928) established with Trolox and expressed as μmol Trolox Equivalents (TE) per gram of dry weight (DW). The relative ORAC value was calculated using the formula: [(AUC_sample_-AUC_black_)/(AUC_Trolox_-AUC_black_)] (molarity of Trolox/molarity of sample).

### 2.9 Free radical scavenging capacity assay

The free radical scavenging capacity was determined using a modified method described in a previous study (Liang, Chang, Liang, Hung, & Hsieh, 2014). One milliliter of the crude extract from section 2.8 was combined with 5 mL of 0.1 mmol/L DPPH ethanol solution, shaken, and allowed to react in the dark at room temperature for 20 min. Absorbance was measured at 517 nm. The DPPH radical scavenging capacity was calculated using the following formula: DPPH radical scavenging capacity = (1 - absorbance of sample/absorbance of blank) × 100 %.)

### 2.10 Ferric reducing antioxidant power (FRAP) assay

The FRAP value was measured using a modified method described in a previous study (Benzie & Strain, 1996). One milliliter of the crude extract from section 2.8was combined with 3 mL of FRAP working solution, shaken thoroughly, and allowed to react in the dark at 37 °C for 30 min. Absorbance was measured at 593 nm. A standard curve was established using Trolox, and the FRAP value was calculated accordingly. The FRAP value was expressed as TE per 100 g of DW. Total antioxidant capacity is calculated using the following formula: (A-A_0_)/(A_max_-A_0_) × 100. A_0_ refers to the A_593_ nm value measured without test sample; A refers to the A_593_ nm value measured when the test sample is added; A_max_ refers to the maximum value of Trolox measured in the experiment.

### 2.11 Metal ion chelating capacity (MCC) assay

MCC was determined using a modified method described in a previous study (Decker & Welch, 1990). One milliliter of the crude extract from section 2.8 was combined with 3.7 mL of methanol, 0.1 mL of 2 mmol/L FeCl_2_•4H_2_O solution, and 0.2 mL of 2.5 mmol/L ferrozine solution, and the mixture was incubated in the dark for 10 min. Absorbance was then measured at 562 nm. MCC was calculated using the following formula: MCC = (1 − absorbance of sample/absorbance of blank) × 100 %.

### 2.12 Metabolomic analysis

The barley samples used for metabolomic analysis of differences in seed coat color include three groups: white, blue, and black. The white group consists of a mixture of three untreated white barley varieties—Xila22, Zangqing2000, and Gamuguori—blended in equal proportions. The blue group is composed of three untreated blue barley varieties—Youxi, Quduigamu, and Garu—mixed in equal proportions. The black group consists of three untreated black barley varieties—Sagui, Qujia, and Xiongzhang—blended in equal proportions. For the metabolomic analysis of differences arising from different cooking methods, the barley samples include three groups: control, frying, and baking. The control group is a mixture of three untreated black barley varieties—Sagui, Qujia, and Xiongzhang—blended in equal proportions. The frying group is prepared by mixing these same three black barley varieties in equal proportions, followed by frying according to the method outlined in section 2.2. The baking group is composed of the same three black barley varieties mixed in equal proportions and baked according to the procedure described in section 2.2. Whole barley grains were ground into a fine powder using a freeze mill, and ∼ 50 mg of this powder was weighed and extracted with 1 mL of 80 % methanol containing 0.1 % formic acid to enhance extraction efficiency. The mixture was vortexed for 5 min and sonicated at 4 °C for 30 min. After centrifugation at 14816*g* for 10 min, the supernatant was collected and filtered through a 0.22-μm membrane filter for analysis. Metabolite profiling was performed using a UHPLC system (Thermo Fisher Scientific, Waltham, MA, USA) coupled with a Q Exactive™ Plus mass spectrometer (Thermo Fisher Scientific). Chromatographic separation was achieved with a Hypersil GOLD™ C_18_ column (100 mm × 2.1 mm, 1.9 μm) at a flow rate of 0.3 mL/min. The mobile phase consisted of solvent A (0.1 % formic acid in water) and solvent B (0.1 % formic acid in acetonitrile). The gradient elution program was as follows: 0–2 min, 2 % B; 2–5 min, 2–25 % B; 5–15 min, 25–95 % B; 15–18 min, 95 % B; 18–18.5 min, 95–2 % B; and 18.5–20 min, 2 % B. The injection volume was 2 μL. Mass spectrometry data were acquired in both positive and negative ion modes with a spray voltage of 3.5 kV and a capillary temperature of 320 °C. The mass range was set to 100–1500 *m*/*z*, and data-dependent acquisition was employed to capture the top 10 most abundant ions for MS/MS fragmentation. Raw data files were processed using Compound Discoverer™ software (Thermo Fisher Scientific) for peak detection, alignment, and quantification. Metabolites were identified by matching against online databases, such as HMDB, METLIN, and KEGG.

### 2.13 Statistical analyses

SAS 9.4 (SAS Inc., Chicago, IL, USA) was used for the statistical analysis of the results. All treatments were performed in triplicate, and the data were expressed as the mean ± standard deviation. Multiple comparisons were performed using Tukey's HSD method with statistical significance set at *P* < 0.05. Multivariate statistical analyses, including principal component analysis (PCA) and orthogonal partial least squares discriminant analysis (OPLS-DA), were performed using SIMCA-P software (Umetrics, Umeå, Sweden).

## 3 Results and analysis

### 3.1 Comparative analysis of phytochemical profiles and antioxidant activities in grain varieties with different seed coat colors

The phytochemical profiles and antioxidant activities of grain varieties with different seed coat colors (white, blue, and black) were comprehensively analyzed (Table 1). Comprehensive profiling of phytochemicals and antioxidant activities across white-, blue-, and black-grained varieties revealed striking color-dependent trends (Table 1). Anthocyanin content, quantified as cyanidin-3-glucoside, was markedly elevated in black-grained cultivars, with the highest-performing variety exceeding the lowest (white-grained) by over 13-fold. Proanthocyanidins followed an analogous pattern, with black varieties accumulating levels up to 2.4 times greater than their white counterparts. Similarly, total flavonoids and phenolic compounds—assessed as rutin and gallic acid equivalents, respectively—peaked in the black-grained group, demonstrating 1.6- to 1.5-fold increases relative to blue- and white-seeded varieties. Intriguingly, bound phenolics were most abundant in a white-grained accession, contrasting with the predominance of free phenolics in black varieties.

**Table 1:** Analysis of antioxidant components and activities among barley varieties with white, blue, and black seed coat colors.

Seed Coat Color	Variety Name	Anthocyanins (as Cyanidin- 3-Glucoside) mg/100 g DW	Proanthocyanidins (mg/100 g DW)	Total Flavonoids (as Rutin) (mg/100 g DW)	Total Phenolics (as Gallic Acid) (mg/100 g DW)	Free Phenolics (as Gallic Acid) (mg/100 g DW)	Bound Phenolics (as Gallic Acid) (mg/100 g DW)	ORAC (μmTE/g FW)	DPPH(%)	FRAP (TE/100 g DW)	MCC(%)
White	Xila22	7.65 ± 0.20 g	81.14 ± 2.38c	313.89 ± 6.86fe	323.14 ± 9.14c	164.48 ± 4.65e	158.66 ± 4.49b	79.42 ± 2.25d	39.70 ± 3.22fe	1941.84 ± 148.44d	26.91 ± 3.03 cd
White	Zangqing2000	17.38 ± 0.30d	69.85 ± 2.14d	369.10 ± 10.69d	377.12 ± 15.31b	199.50 ± 8.10d	177.62 ± 7.21a	83.35 ± 1.89d	37.66 ± 5.25fe	1220.60 ± 201.17e	25.62 ± 2.90d
White	Gamuguori	11.17 ± 0.22f	59.04 ± 2.04e	332.21 ± 11.81e	330.26 ± 8.90c	185.94 ± 5.01d	144.33 ± 3.89 cd	81.18 ± 2.19d	26.58 ± 4.10f	1606.19 ± 262.78ed	32.03 ± 0.90cbd
**White**	verage of varieties	**12.07 ± 4.03C**	**70.01 ± 9.29C**	**338.40 ± 25.05B**	**343.51 ± 26.57B**	**183.30 ± 15.66C**	**160.20 ± 14.66 A**	**81.32 ± 2.66C**	**34.65 ± 7.18C**	**1589.55 ± 361.51C**	**28.19 ± 3.71C**
Blue	Youxi	14.95 ± 0.36e	88.03 ± 2.66b	411.89 ± 9.13c	314.27 ± 7.48c	193.59 ± 4.61d	120.68 ± 2.87e	92.69 ± 3.76c	54.85 ± 6.75 dc	3129.20 ± 414.73cb	34.68 ± 4.22cb
Blue	Quiduigamu	8.43 ± 0.21 g	70.51 ± 1.46d	327.23 ± 6.73e	361.72 ± 15.24b	188.46 ± 7.94d	173.26 ± 7.30a	93.68 ± 3.09c	44.93 ± 6.65de	3263.14 ± 416.98cb	43.81 ± 3.97 A
Blue	Garu	17.87 ± 0.33d	88.43 ± 3.06b	295.33 ± 9.74f	362.73 ± 9.15b	227.79 ± 5.75cb	134.93 ± 3.40d	95.18 ± 2.46c	65.23 ± 8.99bc	2932.47 ± 473.69cb	35.56 ± 2.76b
**Blue**	verage of varieties	**13.75 ± 3.96B**	**82.32 ± 8.72B**	**344.82 ± 49.94B**	**346.24 ± 25.20B**	**203.28 ± 18.55B**	**142.96 ± 22.75 B**	**93.85 ± 3.31B**	**55.00 ± 11.21B**	**3108.27 ± 456.65B**	**38.02 ± 5.53 A**
Black	Sagui	42.06 ± 0.92c	81.75 ± 2.21c	437.64 ± 12.24b	377.06 ± 14.84b	234.53 ± 9.23b	142.53 ± 5.61 cd	96.01 ± 6.49c	79.60 ± 7.37a	3506.28 ± 102.57b	33.02 ± 4.98cbd
Black	Qujia	46.89 ± 0.82b	74.27 ± 1.51d	410.64 ± 15.81c	387.23 ± 10.01b	217.24 ± 5.62c	169.99 ± 4.40a	103.89 ± 2.55b	75.75 ± 7.63ba	2808.50 ± 97.33c	34.42 ± 3.83 cb
Black	Xiongzhang	102.38 ± 2.25a	142.42 ± 5.18a	478.12 ± 13.65a	477.59 ± 13.17a	324.28 ± 8.94a	153.31 ± 4.23cb	117.39 ± 3.24a	82.98 ± 5.59a	4523.43 ± 164.51a	31.67 ± 2.07cbd
**Black**	verage of varieties	**63.78 ± 27.41 A**	**99.48 ± 30.70 A**	**442.13 ± 31.05 A**	**413.96 ± 46.97 A**	**258.68 ± 47.61 A**	**155.28 ± 12.27 A**	**105.76 ± 9.88 A**	**79.44 ± 7.53 A**	**3612.74 ± 715.20 A**	**33.04 ± 3.98B**

Note:Different uppercase letters within the same column indicate significant differences among mixed samples with different seed coat colors while different lowercase letters indicate significant differences among individual varieties.

Four complementary antioxidant assays consistently ranked black-grained cultivars as possessing the highest radical scavenging and reducing capacity. Oxygen radical absorbance capacity (ORAC) and ferric reducing antioxidant power (FRAP) values in the leading black variety surpassed those of the lowest-performing white variety by 48 % and ≥ 2-fold, respectively. DPPH radical inhibition further corroborated this hierarchy, with the darkest-pigmented variety exhibiting >3-fold greater activity than the least active white accession. Notably, metal chelation capacity diverged from this trend, with blue-grained specimens outperforming both darker- and lighter-colored counterparts. These results collectively highlight the superior phytochemical composition and antioxidant activity of black grain varieties, particularly Xiongzhang, in comparison to blue and white varieties. The significant variations in phytochemical content and antioxidant capacity suggest that black grains offer enhanced health benefits, warranting further investigation into their bioavailability and physiological effects. These findings underscore the potential of incorporating these varieties into dietary regimens to leverage their health-promoting properties.

### 3.2 Distinct metabolic pathways and phytochemical profiles in highland barley varieties differentiated by bran color

#### 3.2.1 Metabolomic profiling of highland barley grains differentiated by bran color

**Fig. 1:**
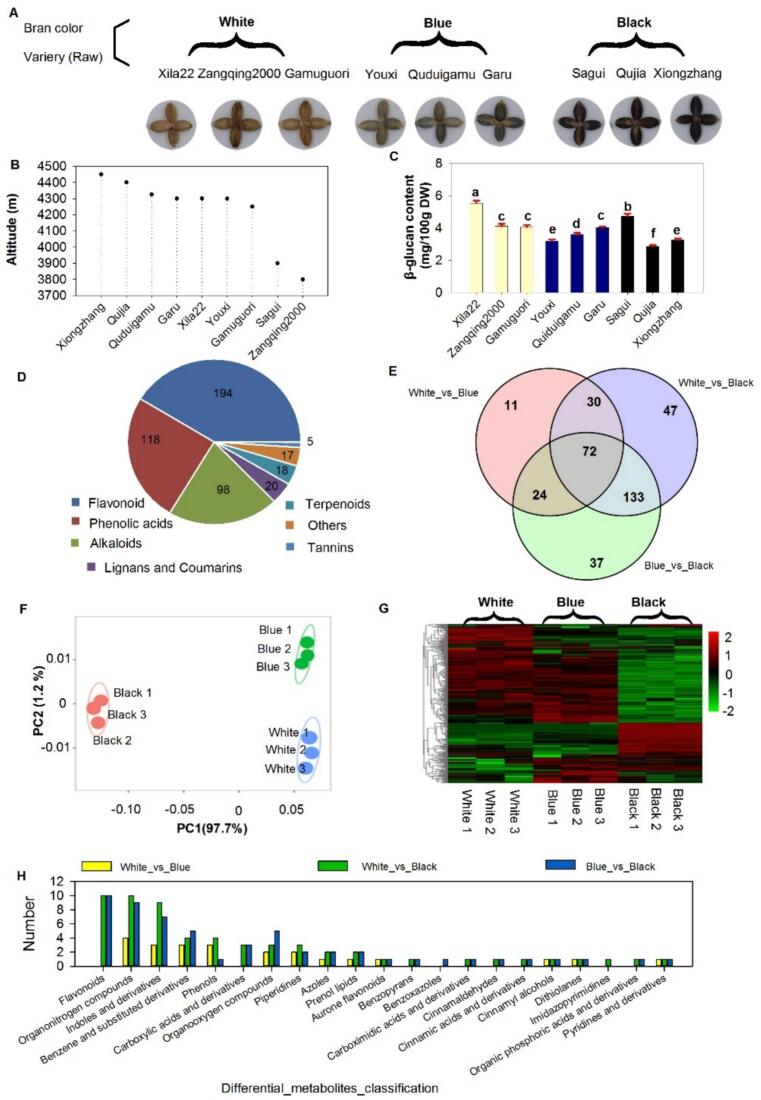
Comprehensive analysis of *β*-glucan content and phytochemical diversity in different bran-colored barley varieties. (A) Visual representation of barley varieties categorized by bran colors. (B) Altitude distribution of highland barley. (C) *β*-Glucan content across the barley varieties, with significant differences denoted by different letters (*P* < 0.05). (D) Pie chart showing mean values of the major phytochemicals identified in barley varieties. (E) A Venn diagram illustrating the overlap of differentially expressed metabolites among white, blue, and black barley varieties. Intersections represent shared metabolites among the comparisons: White_vs_Blue, White_vs_Black, and Blue_vs_Black. (F) Principal component analysis differentiating the barley varieties based on phytochemical composition, with distinct clustering observed for white, blue, and black bran colors. (G) Heatmap illustrating the relative abundance of identified phytochemicals across the barley varieties, with color intensity indicating the concentration levels (red = high, green = low). (H) Classification of differential metabolites across white, blue, and black varieties. White1–3, Blue1–3, and Black1–3 represent biological replicates of white-, blue-, and black-hulled highland barley, respectively. (For interpretation of the references to color in this figure legend, the reader is referred to the web version of this article.)

**Fig. 2:**
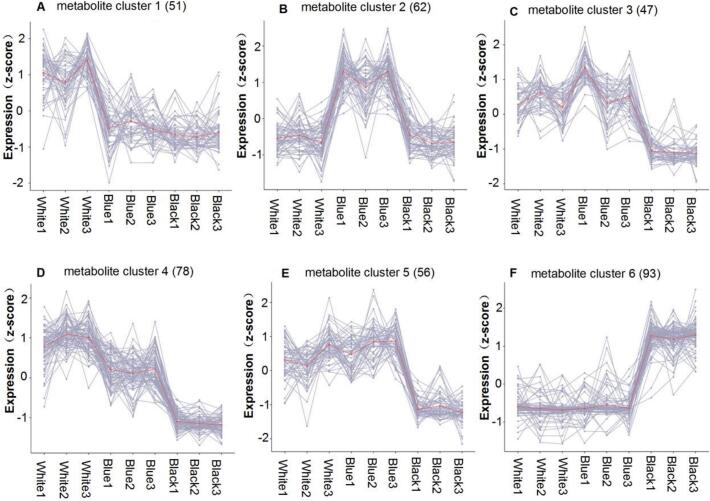
Metabolite clusters in barley varieties of different seed colors. (A) Cluster 1 (51 metabolites), (B) Cluster 2 (62 metabolites), (C) Cluster 3 (47 metabolites), (D) Cluster 4 (78 metabolites), (E) Cluster 5 (56 metabolites), (F) Cluster 6 (93 metabolites). Each line represents a metabolite, with red lines indicating the average expression profile within each cluster. The x-axis shows the comparisons between varieties, and the y-axis indicates the z-score normalized expression levels. White1–3, Blue1–3, and Black1–3 represent biological replicates of white-, blue-, and black-hulled highland barley, respectively. Detailed information for all metabolites in the six clusters is provided in the Supplementary Information. (For interpretation of the references to color in this figure legend, the reader is referred to the web version of this article.)

Highland barley varieties were classified into three groups based on bran color: white, blue, and black (Fig. 1A). The altitudinal distribution (Fig. 1B) presents the altitude of the different varieties, ranging from 3700 m to 4300 m. Xiongzhang is cultivated at the highest altitude, while Zangqing2000 is grown at the lowest. The *β*-glucan content (Fig. 1C) was notably higher in white-bran varieties, with Xila22 exhibiting the highest concentration. β-glucan content variation among the nine barley varieties showed no systematic association with seed coat pigmentation. While some black-grained varieties contained relatively low β-glucan levels, the black variety Sagui accumulated higher concentrations than its white-grained counterpart Zangqing2000, demonstrating that seed color does not predict polysaccharide content. Comprehensive metabolomic analysis of whole-grain highland barley identified 470 distinct metabolites. Metabolite profiling revealed flavonoids as the predominant class (41 %), followed by phenolic acids (25 %) and alkaloids (21 %), collectively representing the major secondary metabolites identified (Fig. 1D). The Venn diagram (Fig. 2E) delineates the overlap of differentially expressed metabolites among white, blue, and black barley varieties, revealing 72 metabolites shared across all comparisons. Notably, flavonoids and alkaloids collectively accounted for 82 % of the differentially abundant metabolites identified. The 72 metabolites common across all comparisons suggest universally affected core metabolic pathways, while the unique metabolites may represent pathways selectively modulated under specific conditions. Principal component analysis (Fig. 1F) demonstrated clear clustering based on bran color, highlights clear differences in the biochemical composition of the varieties based on bran color. The black variety showed clear separation from the white and blue groups. The heatmap (Fig. 1G) displays the relative abundance of identified phytochemicals in each barley variety, with color intensity indicating the concentration levels. White and blue barley share closely related compositional profiles, whereas black barley exhibits marked divergence. The classification of differential metabolites further confirms that both the number and diversity of metabolites are significantly lower in the White_vs_Blue comparison (Fig. 1H). Notably, in the White_vs_Black comparison, black barley is distinguished by a higher abundance of flavonoids, organonitrogen compounds, and indole derivatives, which are present in significantly lower quantities—or entirely absent—in the White_vs_Blue comparison.

#### 3.2.2 Comparative metabolomic profiling of barley varieties with white, blue, and black seeds

The shared and unique metabolites identified in the Venn diagram (Fig. 1E) emphasize the intricate differences among barley grains of different colors. Metabolites associated with highland barley seed coat color were clustered into six well-defined groups based on their differential accumulation patterns. Cluster 1 (Fig. 2A) exhibited elevated metabolite expression in white barley, primarily comprising 17 phenolic acids, 13 flavonoids, and 12 alkaloids. Cluster 2 (Fig. 2B) showed significantly higher metabolite accumulation in blue barley compared to other cultivars, with flavonoids dominating the metabolic profile (37 compounds, 60 % of total detected metabolites). Metabolic profiling revealed similar patterns across clusters 3–5 (Fig. 2C-E), with a predominant downregulation observed in black highland barley. Together, these three clusters comprised 181 metabolites, including 56 flavonoids (30.9 %), 54 alkaloids (29.8 %), 44 phenolic acids (24.3 %), 9 terpenoids (5.0 %), and 5 lignans/coumarins and tannins (2.8 % each). In contrast, cluster 6 (Fig. 2F) exhibited marked upregulation in black barley, containing 68 flavonoids (73.9 % of cluster metabolites), 11 phenolic acids (12.0 %), 7 lignans/biotin derivatives (7.6 %), 5 alkaloids (5.4 %), and 1 terpenoid (1.1 %). Clusters of metabolites exhibited diverse expression patterns, highlighting the metabolic heterogeneity among these barley varieties.

The observed differential accumulation (both suppression and promotion) of metabolites in specific clusters likely reflects corresponding modulation of key metabolic pathways in highland barley (*Hordeum vulgare* L.). These findings are consistent with previous reports documenting environment-induced metabolic plasticity in cereals (Yu et al., 2020; Zhang et al., 2023). However, the complexity and diversity of these metabolic changes pose significant challenges in establishing definitive pathway associations. To resolve these relationships, we conducted systematic pathway enrichment analysis (Section 3.2.3) with two primary objectives: (i) to identify potential biochemical networks connecting these differentially accumulated metabolites, and (ii) to uncover the mechanistic drivers of phytochemical variation associated with seed coat pigmentation. This approach builds upon established frameworks for metabolic interpretation (Li, Duncan, Li, Huang, & Luo, 2024; Yang et al., 2018) while addressing critical gaps in our understanding of barley's specialized metabolism.

#### 3.2.3 Metabolic pathway enrichment and metabolite network analysis in highland barley varieties with different bran colors

**Fig. 3:**
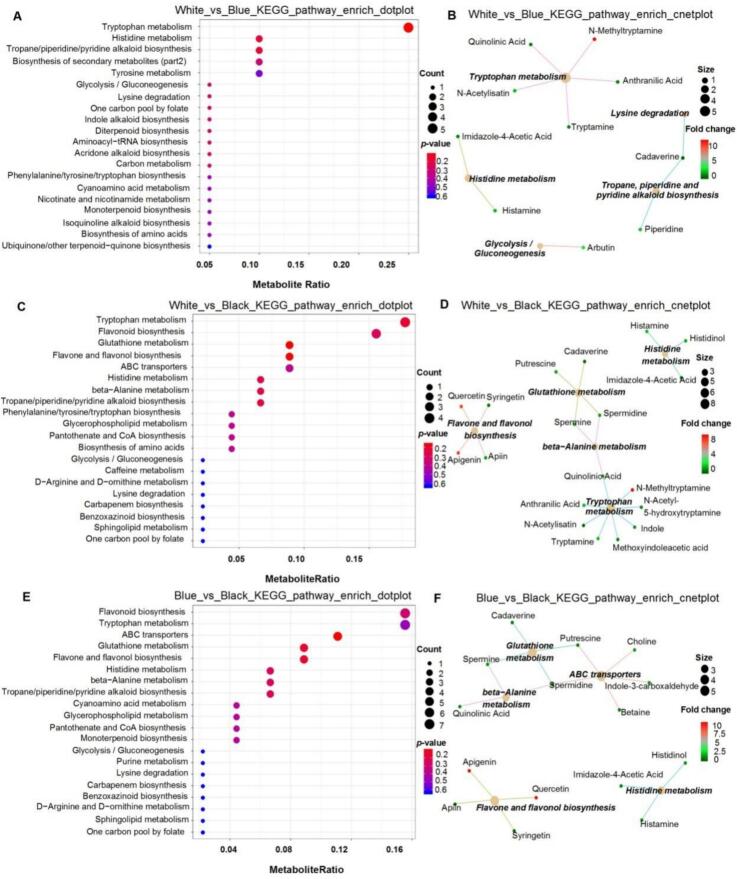
KEGG pathway enrichment analysis of metabolomic differences among white, blue, and black highland barley varieties. (A) Dot plot for White_vs_Blue comparison, highlighting significant enrichment in pathways such as tryptophan metabolism, histidine metabolism, and the biosynthesis of secondary metabolites. (B) Cnetplot for White_vs_Blue, showing the network of enriched pathways and associated metabolites, with key nodes including quinolinic acid and *N*-acetylaspartate. (C) Dot plot for White_vs_Black comparison, with notable enrichment in pathways like flavone and flavonol biosynthesis, and glutathione metabolism. (D) Cnetplot for White_vs_Black, illustrating central metabolites such as putrescine and cadaverine. (E) Dot plot for Blue_vs_Black comparison, identifying significant enrichment in pathways including flavonoid biosynthesis, and tryptophan metabolism. (F) Cnetplot for Blue_vs_Black, featuring key metabolites such as cadaverine and glutathione, highlighting the metabolic distinctions between these barley varieties. (For interpretation of the references to color in this figure legend, the reader is referred to the web version of this article.)

Comparative KEGG pathway enrichment analysis revealed distinct metabolic pathways across white, blue, and black barley varieties. Dot plots (Fig. 3A, C, E) illustrate the enrichment of various metabolic pathways, with metabolite ratios and *p* values indicating the significance of each pathway. In the white versus blue comparison (Fig. 3A), pathways such as tryptophan metabolism, histidine metabolism, and the biosynthesis of secondary metabolites were notably enriched in blue barley. The network plot (Fig. 3B) visualizes these pathways, showing key metabolites, such as quinolinic acid and *N*-acetylisatin, that exhibit significant fold changes. In the white versus black comparison (Fig. 3C), pathways such as flavone and flavonol biosynthesis and glutathione metabolism were significantly enriched in black barley. The network plot (Fig. 3D) highlights crucial metabolites, including quercetin and syringetin, which exhibit substantial fold changes and suggest a pivotal influence on the metabolic profile of black barley. Similarly, the blue versus black comparison (Fig. 3E) revealed pathways such as flavonoid biosynthesis and lysine degradation enriched in black barley. The network plot (Fig. 3F) illustrates these pathways, highlighting significant changes in key metabolites such as cadaverine and glutathione.

These findings highlight the influence of bran color on the metabolic diversity of highland barley. The enriched pathways and key metabolites identified offer valuable insights into the biochemical mechanisms underlying the distinct nutritional and functional properties of white, blue, and black barley varieties, providing potential targets for breeding programs to optimize nutritional and functional properties.

### 3.3 Impact of cooking methods on β-glucan content and metabolomic pathway alterations in black highland barley

Black highland barley is notable for its high content of anthocyanins and polyphenols, which are bioactive compounds known for their potent antioxidant properties. These attributed not only enhance its nutritional profile but also suggest its potential for development as a functional food product. Given the promising bioactivity of black barley, particularly its antioxidative capacity, this study aims to conduct a comprehensive investigation into the metabolic effects associated with its consumption. Through this research, we seek to elucidate the specific metabolic pathways influenced by black barley, contributing to a deeper understanding of its role in health promotion and potential applications in the food industry.

#### 3.3.1 Effects of cooking methods on β-glucan content and metabolite profiles in black highland barley

Given the high content of flavonoid-rich bioactive compounds in black-hulled highland barley and the increasing consumer preference for dark-colored foods, black barley is more extensively utilized in the development of health-oriented products compared to white and blue barley varieties. Therefore, black highland barley were selected as the subject of this study to investigate the effects of cooking methods on its metabolomic profile.

**Fig. 4:**
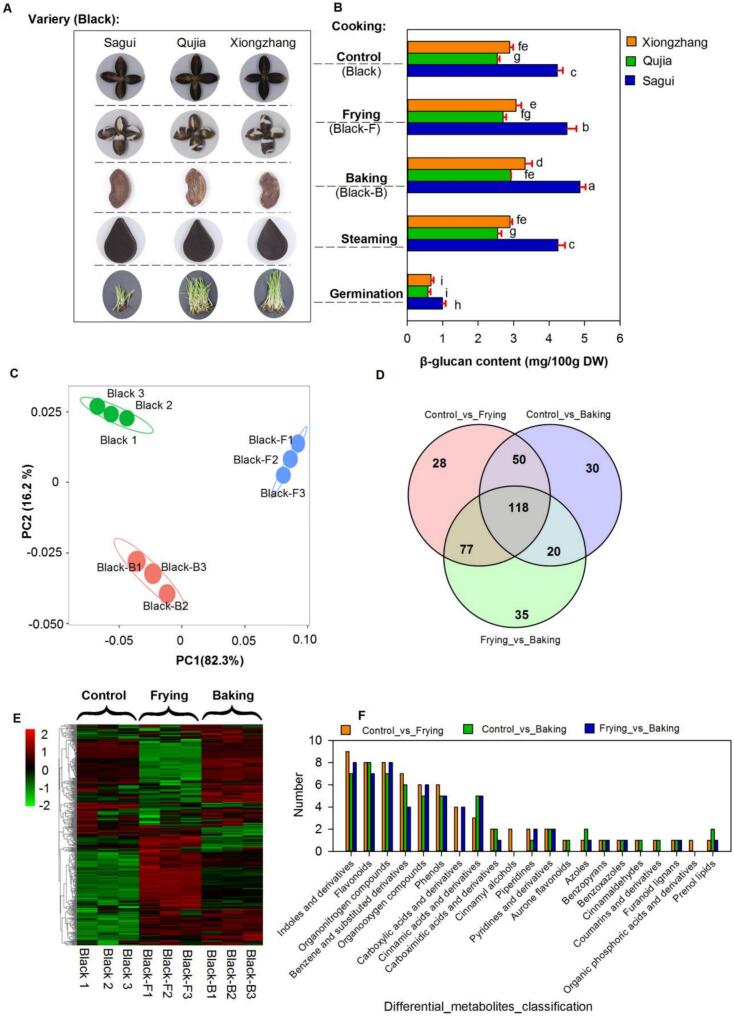
Differential impact of cooking methods on black highland barley. (A) Visual representation of three black highland barley varieties (Sagui, Qujia, and Xiongzhang) subjected to various cooking treatments: control, frying, baking, steaming, and germination. (B) Quantification of *β*-glucan content across different cooking methods and barley varieties. Statistical significance (*p* < 0.05) is indicated by different letters above bars. (C) Principal component analysis of metabolite profiles showing clear separation by cooking method and variety, with PC1 and PC2 explaining 98.5 % of the variance. (D) Venn diagram showing the overlap of differentially expressed metabolites among Control_vs_Frying, Control_vs_Baking, and Frying_vs_Baking comparisons, highlighting a core set of 118 metabolites consistently affected across all treatments and unique metabolite sets for each comparison.(E) Heatmap illustrating the expression levels of differentially expressed metabolites across control, frying, and baking treatments, with color intensity representing log2-transformed expression levels. Three biological replicates each were analyzed for raw (Black1–3), fried (Black-F1–3), and baked (Black-B1–3) black-hulled highland barley.

The metabolic profiles of three black highland barley varieties (Sagui, Qujia, and Xiongzhang) were analyzed under various cooking treatments, including control (raw), frying, baking, and steaming (Fig. 4A). Since β-glucan is a key bioactive compound in barley, special attention should be given to its protection during processing. Sprouting is commonly used as a pre-cooking method to enhance the bioactive components and reduce anti-nutritional factors in grains. In this study, it is employed as a comparative treatment method to evaluate other processing techniques. Significant changes in β-glucan content were observed across these cooking treatments (Fig. 4B), with frying and baking resulting in a notable increase in β-glucan levels, by approximately 6 % and 15 %, respectively. In contrast, steaming did not increase the β-glucan content in any of the three barley varieties. Germination led to a significant reduction in β-glucan content, which was only around 23 % of that in the control group. Based on optimization of processing methods, it was determined that frying and baking were effective in enhancing the β-glucan content in barley. Therefore, the metabolic changes in black barley following frying and baking treatments were the main focus of the subsequent research.

Principal component analysis (PCA) of metabolite profiles revealed clear separation by both cooking method and variety (Fig. 4C). PC1 and PC2 accounted for 98.5 % of the variance, indicating that distinct metabolic changes were induced by the different cooking treatments. Biological replicates for each treatment—Black1–3 (raw), Black-F1–3 (frying), and Black-B1–3 (baking)—showed reproducibility across conditions, supporting the robustness of the experimental setup. The Venn diagram (Fig. 4D) further highlighted the overlap of differentially expressed metabolites across the cooking treatments, identifying a core set of 118 metabolites that were consistently affected across all conditions. Unique sets of metabolites were found in each comparison (Control_vs_Frying: 87 metabolites; Control_vs_Baking: 92; Frying_vs_Baking: 68), emphasizing the distinct metabolic shifts induced by each cooking method. The heatmap (Fig. 4E) illustrated the expression levels of these metabolites, with color intensity representing the log2-transformed expression values. Comparative analysis revealed substantial metabolic alterations induced by processing methods. The frying treatment caused pronounced metabolic shifts relative to untreated controls, while baking induced more moderate changes. Notably, only 15 % of detected metabolites maintained consistent expression patterns between fried and baked samples, indicating distinct biochemical impacts for each thermal processing method.

The classification of differentially expressed metabolites in black highland barley subjected to various cooking methods (control, frying, and baking) revealed distinct metabolic alterations across treatments (Fig. 4F). Among the metabolite classes, indoles and derivatives, organonitrogen compounds, and flavonoids exhibited the highest number of differentially expressed metabolites across all comparisons. Specifically, the Control_vs_Frying (orange) and Control_vs_Baking (green) comparisons both showed substantial changes in these categories, with a slightly higher number of differentially expressed metabolites in the Control_vs_Frying comparison.

Further analysis revealed that, overall, the number and diversity of differential metabolites were greater in the Control_vs_Frying comparison compared to the Control_vs_Baking comparison. This suggests that more metabolites responded more strongly to frying. Other classes, such as azoles and benzopyrenes, exhibited minor changes, contributing to a more specific metabolic shift influenced by frying and baking methods. These findings underscore the complexity of metabolic changes induced by cooking methods, with distinct metabolite classes being preferentially altered depending on the treatment applied to black highland barley. These results suggest that cooking methods significantly modulate the metabolic landscape of barley, with implications for its nutritional and bioactive properties.

#### 3.3.2 Metabolomic profiling of black highland barley reveals pathway-specific alterations induced by different cooking methods

**Fig. 5:**
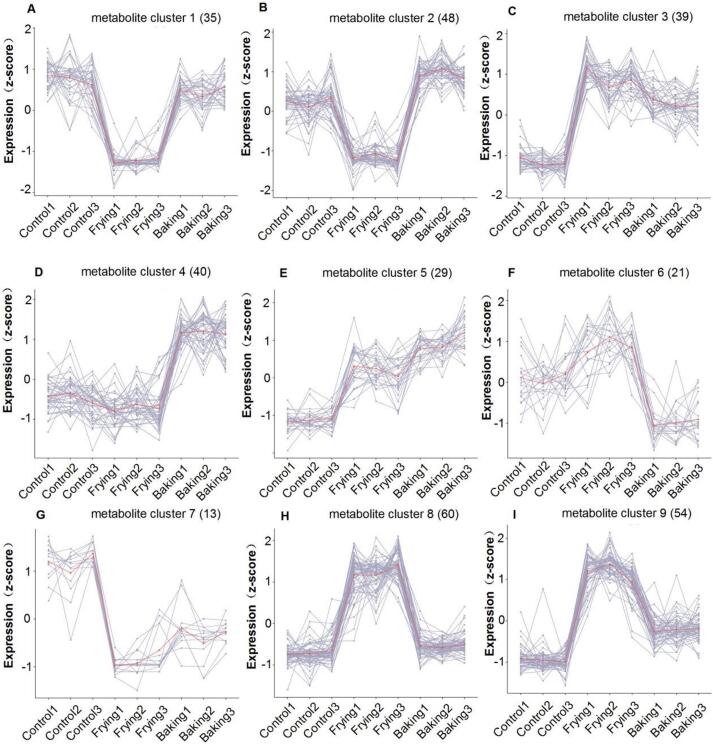
Metabolic responses of black highland barley to control, frying, and baking treatments. (A-I) Hierarchical clustering identified 9 distinct metabolite clusters based on their expression profiles (z-scores) across control, frying, and baking treatments: (A) Cluster 1 (35 metabolites), (B) Cluster 2 (48 metabolites), (C) Cluster 3 (39 metabolites), (D) Cluster 4 (40 metabolites), (E) Cluster 5 (29 metabolites), (F) Cluster 6 (21 metabolites), (G) Cluster 7 (13 metabolites), (H) Cluster 8 (60 metabolites) and (I) Cluster 9 (54 metabolites), Control1–3, Frying1–3, and Baking1–3 represent the three biological replicates for the control, frying, and baking groups, respectively. Detailed information for all metabolites in the nine clusters is provided in the Supplementary Information.

Metabolic profiling of black highland barley via hierarchical clustering revealed nine distinct metabolite clusters with treatment-specific modulation patterns, with frying inducing the most pronounced metabolic reorganization (Fig. 5). Cluster 1, comprising 35 metabolites, showed significant downregulation in fried samples, predominantly consisting of flavonoids (51.4 %) and alkaloids (17.1 %). In Cluster 2, which included 48 metabolites, consistent suppression was observed in frying, with alkaloids (45.8 %) and flavonoids (37.5 %) as the major components. Notably, Cluster 3, with 39 metabolites, demonstrated coordinated upregulation in both fried and baked samples, featuring phenolic acids (46.2 %) and flavonoids (30.8 %), indicating thermal activation of phenylpropanoid pathways. Cluster 4, consisting of 40 metabolites, revealed a baking-specific elevation, primarily flavonoids (55.0 %), suggesting the presence of Maillard reaction products. Clusters 5 and 6 exhibited treatment-divergent regulation, with Cluster 6 showing upregulation of flavonoids in frying but suppression in baking. Cluster 7, enriched in control metabolites (61.5 % flavonoids), displayed degradation upon cooking. Clusters 8 and 9, in contrast, demonstrated frying-specific upregulation of phenolic acids (41.7 %) and alkaloids (26.7 %), highlighting that frying preferentially alters specialized metabolism by reducing health-promoting flavonoids while inducing potentially bioactive alkaloids and phenolic acids. The baking-specific flavonoid enrichment observed in Cluster 4 suggests that milder thermal processing better preserves nutritional components.

#### 3.3.3 Metabolomic pathway alterations in highland barley induced by different cooking methods

**Fig. 6:**
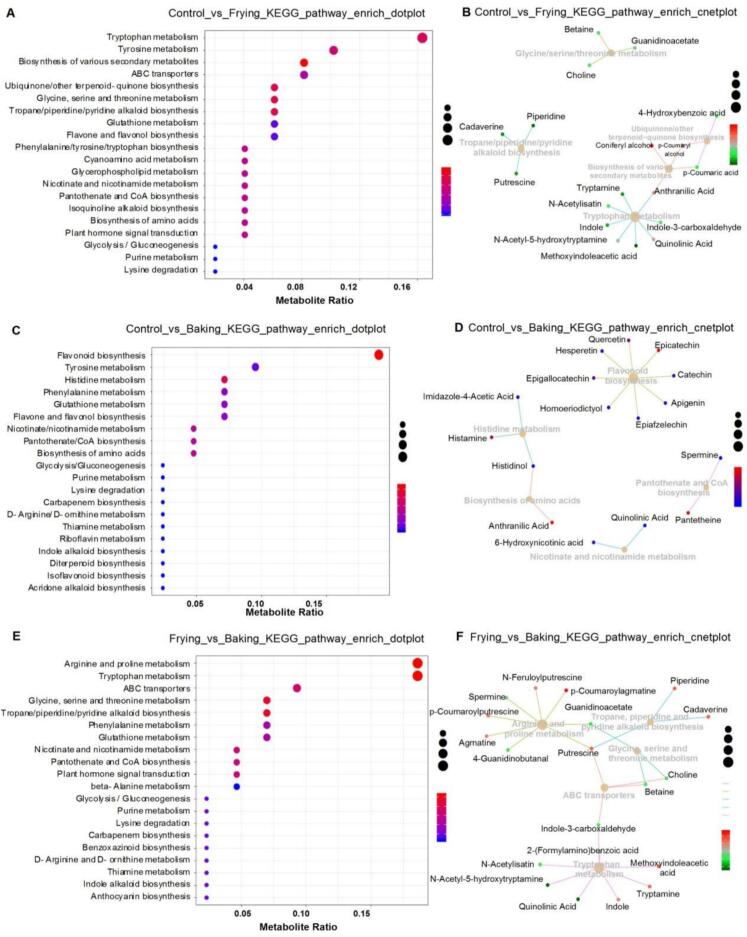
KEGG pathway enrichment analysis of metabolomic changes in highland barley subjected to frying and baking treatments. (A) KEGG pathway enrichment dot plot for Control_vs_Frying. The plot highlights the most significantly enriched pathways, with the metabolite ratio and *p*-value indicated by dot size and color, respectively. (B) Cnetplot for Control_vs_Frying, showing the network of enriched pathways and associated metabolites. Key metabolites like betaine, guanidinoacetate, and choline are centrally involved in the modulated pathways. (C) KEGG pathway enrichment dot plot for Control_vs_Baking. Significant pathways include flavonoid biosynthesis, tyrosine metabolism, and histidine metabolism, as indicated by the dot plot. (D) Cnetplot for Control_vs_Baking, visualizing the network of enriched pathways and metabolites. Central metabolites such as epicatechin and anthranilic acid play pivotal roles in the baking-induced metabolic shifts. (E) KEGG pathway enrichment dot plot for Frying_vs_Baking. Key pathways like arginine and proline metabolism, tryptophan metabolism, and flavone and flavonol biosynthesis are significantly enriched. (F) Cnetplot for Frying_vs_Baking, depicting the network of enriched pathways and their associated metabolites. Critical nodes include putrescine and agmatine, indicating substantial modulation by the cooking methods.

This study investigated the effects of control, frying, and baking treatments on the metabolic pathways of black highland barley using KEGG pathway enrichment analysis. Dot plots (Fig. 6A, C, and E) highlight the most significantly enriched pathways, while cnet plots (Fig. 6B, D, and F) depict the network of metabolite-pathway interactions. In the Control_vs_Frying comparison (Fig. 6A, B), significant enrichments were observed in pathways such as tryptophan metabolism, tyrosine metabolism, and secondary metabolite biosynthesis, with central metabolites including betaine, guanidinoacetate, and choline playing pivotal roles. In the Control_vs_Baking comparison (Fig. 6C, D), pronounced effects were seen in flavonoid biosynthesis, tyrosine metabolism, and histidine metabolism, where metabolites such as epicatechin and anthranilic acid were central to the metabolic shifts. The Frying_vs_Baking comparison (Fig. 6E, F) revealed marked differences in arginine and proline metabolism, tryptophan metabolism, and flavone and flavonol biosynthesis, with putrescine and agmatine identified as key metabolites in these pathways.

The KEGG pathway enrichment analysis demonstrates that different cooking methods profoundly influence the metabolic pathways of black highland barley. Tryptophan and tyrosine metabolism exhibited notable changes in both the Control_vs_Frying and Control_vs_Baking comparisons, suggesting that these amino acid pathways are particularly sensitive to thermal processing. Frying appears to accelerate these metabolic reactions, potentially by disrupting cell structure and promoting certain chemical reactions, thereby leading to increased metabolite production. In comparison, baking exerts a milder effect, enhancing metabolite enrichment while preserving certain nutrients. While baking also promotes flavonoid synthesis and amino acid metabolism, its gentler treatment helps maintain the stability of critical compounds, such as vitamins and pantothenic acid. The lower temperatures during baking may better preserve the integrity of metabolic reactions, minimizing the production of decomposition products. These pathways are crucial for plant stress responses and secondary metabolite synthesis, highlighting the impact of thermal processing on the metabolic profile of barley (Isah, 2019; Jan, Asaf, Numan, Lubna & Kim, 2021). Central metabolites, such as putrescine and agmatine, emerged as key nodes, suggesting their potential as biomarkers for evaluating the metabolic effects of different cooking methods.

## 4 Discussion

### 4.1 Relationship between grain color and bioactive compounds

This study has elucidated the complex relationship between grain color and the accumulation of bioactive compounds in barley, revealing substantial variations in the concentrations of anthocyanins, proanthocyanidins, flavonoids, and phenolic acids among white, blue, and black barley varieties. Field investigations conducted on the Tibetan Plateau identified a distinct vertical zonal distribution of barley grain color: below 3000 m, white grains predominated; between 3000 and 4000 m, the proportion of blue and purple grains significantly increased; and above 4000 m, black grain phenotypes began to appear. This distribution pattern is primarily driven by two major environmental factors: ultraviolet radiation and low oxygen stress. At higher altitudes, ultraviolet radiation is amplified several times compared to lower elevations, which is significantly correlated with the activation of UV receptors. Additionally, hypoxic conditions induced by low oxygen levels promote the upregulation of flavonoid biosynthesis pathways.

These findings are consistent with previous research that suggests lighter-colored grains typically contain lower levels of phenolic compounds (Ge et al., 2021). White barley varieties display markedly lower concentrations of these bioactive compounds, while blue barley varieties exhibit intermediate levels of anthocyanins and phenolics. Notably, black barley, particularly the Xiongzhang variety, displays the highest concentrations of anthocyanins and total phenolics—bioactive compounds known for their potent antioxidant, anti-inflammatory, and anticancer properties (Bendokas et al., 2020; Ma et al., 2014). This reinforces the hypothesis that grain pigmentation is directly correlated with the accumulation of these bioactive compounds. With its higher anthocyanin content, black barley may offer greater antioxidant protection and health benefits than white and blue varieties. These findings underscore the potential of colored barley varieties in developing functional foods and dietary supplements aimed at enhancing health and preventing chronic diseases.

### 4.2 Impact of cooking methods on β-glucan content in black highland barley

β-glucan is a soluble fiber with various health benefits, including cholesterol reduction and improved glycemic control (J. Bai et al., 2019). The impact of cooking methods on β-glucan content in grains primarily involves changes in solubility, molecular structure, and the extent of loss. Previous studies have shown that boiling increases the solubility of β-glucan, releasing it from the cell wall into the water; however, discarding the cooking liquid leads to a loss of β-glucan. Additionally, during fermentation, some β-glucan may be degraded by microorganisms, resulting in a reduction in its content. Pre-cooking treatments such as sprouting, which involves enzymatic activity and germination, have been found to reduce β-glucan levels due to the activation of β-glucanase enzymes, which break it down into smaller units (Hübner & Arendt, 2013).

This study revealed that frying and baking significantly increased β-glucan levels. The observed increase is attributed to the breakdown of cell walls, which releases β-glucan and enhances its bioavailability (Y. Zhang et al., 2023). Interestingly, steaming did not significantly increase β-glucan levels, likely because β-glucan dissolves in water and is lost during the cooking process. These findings underscore the importance of selecting appropriate cooking methods to retain or enhance the nutritional quality of black highland barley. The role of β-glucan in lowering cholesterol and regulating blood sugar highlights the need to optimize cooking techniques for dietary planning and functional food development.

When baking or roasting, high temperatures may damage the molecular structure of β-glucan, reducing its functionality. It is crucial to control temperature and cooking time to minimize the degradation of β-glucan and maximize its health benefits. Further studies should explore optimal cooking conditions that preserve or enhance β-glucan content in barley and other grains (Y. Li, Lu, Liao, & Liu, 2023).

### 4.3 Secondary metabolic pathway alterations induced by cooking methods

The secondary metabolome of highland grains primarily includes bioactive compounds such as phenolic compounds, flavonoids, alkaloids, and terpenoids. These substances not only influence the nutritional quality of grains but are also closely linked to human health. Different cooking methods significantly alter the composition and content of these secondary metabolites through mechanisms such as thermal effects, oxidation, and hydrolysis. Metabolomic profiling revealed that different cooking methods induce significant, pathway-specific alterations in highland barley. Thermal processes, such as frying and baking, were found to enhance the biosynthesis of flavonoids and phenolic acids, consistent with previous studies showing that cooking can enhance the release and bioavailability of these compounds (L. Zhang, Cui, Ma, Han, & Han, 2023; Wu et al., 2022; Gu, Jin, Schwarz, Rao, & Chen, 2023).

Principal component analysis and heatmap analyses revealed distinct metabolic profiles associated with each cooking method. These changes highlight the transformative effects of thermal and enzymatic treatments on the metabolomic landscape of barley (Raj et al., 2023). For example, increased levels of flavonoids and phenolic acids in fried and baked barley contribute to enhanced antioxidant activity and other health benefits. Moreover, cooking methods affected pathways related to amino acid metabolism, lipid metabolism, and carbohydrate metabolism, reflecting a broad spectrum of biochemical changes (H. Huang et al., 2024). Such changes influence the nutritional and functional properties of barley. For instance, alterations in amino acid metabolism pathways may affect protein quality and the bioavailability of essential amino acids. Understanding how thermal processes alter the nutritional value of barley can facilitate the optimization of food processing techniques and the development of functional foods with targeted health benefits. Insights from metabolomic profiling further support the creation of innovative food products designed to enhance health and prevent diseases.

## 5 Conclusions

This study provides a comprehensive understanding of how different cooking methods affect the *β*-glucan content and metabolomic profiles of black highland barley. These findings indicate that frying and baking enhances *β*-glucan levels, while germination leads to a marked reduction. Metabolomic profiling further revealed pathway-specific alterations caused by different cooking methods, particularly in the biosynthesis of flavonoids and phenolic acids. These results underscore the importance of selecting appropriate cooking methods to maximize the nutritional and health benefits of barley. Furthermore, the analysis of grain color and its relationship with bioactive compounds revealed significant variations in anthocyanin, flavonoid, and phenolic acid contents across white, blue, and black barley varieties. With its higher levels of these compounds, black barley offers greater antioxidant protection and health benefits, establishing its potential as a key ingredient in the development of functional foods. The impact of cooking methods on nutritional quality of barley highlights the need for further research to optimize processing techniques. Future studies should explore the underlying mechanisms driving metabolic changes induced by cooking and their implications for human health. Insights from this research can also inform the creation of new food products with enhanced nutritional profiles and health-promoting properties.

## CRediT authorship contribution statement

**Xinkun Wang:** Writing – original draft, Validation, Software, Methodology, Investigation, Data curation. **Chune Peng:** Writing – original draft, Validation, Software, Methodology, Investigation, Data curation. **Jingwen Fan:** Writing – original draft, Validation, Software, Methodology, Investigation, Data curation, Conceptualization. **Hongyu Si:** Writing – original draft, Validation, Methodology, Investigation, Data curation, Conceptualization. **Sujun Sun:** Writing – original draft, Validation, Methodology, Investigation, Data curation, Conceptualization. **Xiaodan Dong:** Writing – original draft, Validation, Methodology, Investigation, Data curation, Conceptualization. **Hengzhen Wang:** Writing – original draft, Validation, Methodology, Investigation, Data curation, Conceptualization. **Yueming Wang:** Writing – original draft, Validation, Methodology, Investigation, Data curation, Conceptualization. **Peng Deng:** Writing – review & editing, Supervision, Project administration, Funding acquisition. **Lizeng Peng:** Writing – review & editing, Supervision, Project administration, Funding acquisition.

## Declaration of competing interest

The authors declare that they have no known competing financial interests or personal relationships that could have appeared to influence the work reported in this paper. The authors declare no conflict of interest.

## Data Availability

Data will be made available on request.
